# A fluorometric assay to determine the protective effect of glucose-6-phosphate dehydrogenase (G6PD) against a *Plasmodium* spp. infection in females heterozygous for the G6PD gene: proof of concept in *Plasmodium falciparum*

**DOI:** 10.1186/s13104-022-05952-1

**Published:** 2022-02-22

**Authors:** Angela Rumaseb, Jutta Marfurt, Steven Kho, Maria Kahn, Ric N. Price, Benedikt Ley

**Affiliations:** 1 Global and Tropical Health Division, Menzies School of Health Research, Charles Darwin University, Casuarina, PO Box 41096, Darwin, NT 0811 Australia; 2 PATH Diagnostics Group, Seattle, WA USA; 3 Centre for Tropical Medicine and Global Health, Nuffield Department of Medicine, University of Oxford, Oxford, UK; 4 Mahidol‐Oxford Tropical Medicine Research Unit, Mahidol University, Bangkok, Thailand

**Keywords:** Glucose-6-phosphate dehydrogenase (G6PD), G6PD deficiency, Heterozygous females, Flow cytometry, Malaria

## Abstract

**Objective:**

Glucose-6-phosphate dehydrogenase (G6PD) deficiency offers some protection against malaria; however, the degree of protection is poorly described and likely to vary with G6PD genotype and *Plasmodium* species. We present a novel approach to quantify the differential invasion rates of *P. falciparum* between G6PD deficient and normal red blood cells (RBCs) in an ex vivo model. A flow-cytometry based assay was developed to distinguish G6PD deficient and normal, parasitized and non-parasitized RBCs within the same sample. Venous blood collected from a G6PD heterozygous female was infected and cultured ex vivo with a laboratory strain of *P. falciparum* (FC27).

**Results:**

Aliquots of infected blood were assayed at schizont and subsequent synchronized ring stages. At schizont stage, 84.9% of RBCs were G6PD deficient of which 0.4% were parasitized compared to 2.0% of normal RBCs. In the subsequent ring stage, 90.4% of RBCs were deficient and 0.2% of deficient and 0.9% of normal cells respectively were parasitized. The pooled Odds Ratio for a deficient RBC to be parasitized was 0.2 (95% confidence interval: 0.18–0.22, p < 0.001) compared to a normal cell. Further studies are warranted to explore preferential parasitization with different G6PD variants and *Plasmodium* species.

**Supplementary Information:**

The online version contains supplementary material available at 10.1186/s13104-022-05952-1.

## Introduction

Glucose-6-phosphate dehydrogenase (G6PD) deficiency is among the most common enzymopathies worldwide affecting approximately 400 million people [1]. There is a striking overlap between areas with high prevalence of G6PD deficiency and areas that were, or still are, endemic for malaria [2]. Although this suggests an interaction between G6PD enzyme activity and the malaria parasite, a meta-analysis of 30 studies from Africa and Asia provided conflicting evidence, suggesting some protection from *Plasmodium (P.) falciparum* in Africa but not in Asia, and no association between G6PD deficiency and other malaria species [3]. The G6PD gene is located on the X-chromosome, whilst hemizygous males and homozygous females are either G6PD deficient or G6PD normal, heterozygous females carry two copies of the gene and thus have two distinct RBCs populations, in most cases a G6PD deficient and G6PD normal one [4]. In 1969, Luzzatto and colleagues analyzed blood smears collected for microscopic blood film examination from 20 females heterozygous for the G6PD gene with *P. falciparum* infection in Nigeria [5]. Slides were stained using methemoglobin elution to distinguish between G6PD normal and deficient cells and the proportions of infected cells among both types of RBCs were compared. The probability of a G6PD normal RBC being infected with *P. falciparum* was 2 to 80 times higher compared to deficient RBCs [5]. Unfortunately, skilled malaria microscopists are scarce, staining and counting are time-consuming, and precise quantification is challenging. To test the hypothesis that G6PD normal RBCs are more likely to be invaded by *P. falciparum* than G6PD deficient RBCs and to quantify this effect objectively on a larger scale, novel approaches are needed. The aim of this study was to develop a standardized flow-cytometry-based assay to quantify the proportions of infected and uninfected G6PD normal and deficient RBCs in samples from heterozygous females infected with *P. falciparum*.

## Main text

A total of 14 mL of venous blood were collected from a healthy female heterozygous for the G6PD gene. At the time of venepuncture, her G6PD activity was 2.31 U/gHb measured by spectrophotometry (Pointe Scientific, Canton, MI, USA) and 2.15 U/gHb by SD Biosensor (SD Bioline, Gyeonggi-do, South Korea). RBCs were separated from whole blood by centrifugation and suspended in phosphate-buffered saline (PBS; pH 7.4) before being passed through a non-woven filter (Antoshin, Singapore) to deplete host white blood cells (WBCs) present within the sample. Filtered RBCs were resuspended in RPMI 1640 medium with 10% human serum to create a 50% hematocrit suspension and were stored at 4 °C for up to 2 weeks. Aliquots of the RBC suspension were then added to malaria cultures containing the chloroquine (CQ)-sensitive *P. falciparum* strain FC27 (BEI Resources, ATCC Manassas, USA) and maintained using previously published methods [6]. The culture was incubated at 5% hematocrit in a candle jar at 37 °C to adapt to donor’s RBCs. Experiments were done in duplicates on schizont stages and the subsequent ring stage post-synchronization (Additional file 3: Fig. 1) with d-sorbitol (Sigma-Aldrich, Burlington, MA, USA) [7].

White cell depletion allowed continuous culture and the flow cytometric identification of parasites using a DNA dye. For each measurement, a 5% infected RBC suspension was stained for parasite DNA using DRAQ5 (BioLegend, San Diego, CA, USA) at a final concentration of 6.25 µM and incubated in the dark for 20 min at room temperature. After washing the cells twice with PBS, cells were re-suspended in 100 µL PBS and a cytofluorometric assay was performed to differentiate the G6PD activity of individual RBCs based on their differential methemoglobin reduction and sequential reactions products as described previously [8]. The detailed procedure is described in Additional file 1.

Samples were acquired using a dual laser Beckman Coulter Gallios flow cytometer and analyzed using Kaluza version 2.1 (Beckman Coulter, Brea, CA, USA). A minimum of 50,000 events were acquired for each sample and the representative gating strategy is shown in Additional file 4: Fig. 2. G6PD normal and deficient RBC populations were identified in FL1 (533 ± 30 nm bandpass filter); G6PD deficient RBCs were weakly fluorescent from the cyanmethemoglobin product of the assay, whereas G6PD normal RBCs fluoresced strongly as a result of the conversion of oxyhemoglobin to ferryl-hemoglobin in the presence of hydrogen peroxide. The DNA dye DRAQ5 was detected in FL8 (775 long pass filter) and differentiated parasitized (DRAQ5-positive) from non-parasitized (DRAQ5-negative) RBC populations.

Proportions of infected and uninfected G6PD deficient and normal RBCs were compared for each measurement and the odds ratio (OR) and 95% confidence interval (95% CI) were calculated to quantify any protective effect of G6PD deficiency against a *P. falciparum* invasion.

Measurements were done in duplicate at schizont stage and 4 h later at ring stage post-synchronization (Table 1, Additional file 2). At schizont stage, two distinct RBC populations were observed in FL1, with 84.9% of RBCs G6PD deficient and 15.1% G6PD normal. During this stage, 0.4% of deficient RBCs were parasitized, compared to 2.0% of normal RBCs. In the subsequent cycle, the proportion of deficient RBCs increased to 90.4%, 0.2% were parasitized compared to 0.9% of the remaining normal RBCs. In both experiments, deficient RBCs were 5 times less likely to be invaded by the FC27 strain (pooled OR 0.2, 95% CI 0.18 to 0.22), irrespective of whether considering the schizont phase or the subsequent ring stage. The calculated ORs did not differ within or between stages.

**Table 1:** Absolute numbers and proportions of parasitized and non-parasitized G6PD deficient and normal RBCs

Experiment	G6PDn not infected RBCs (% of normal RBC)	G6PDn infected RBCs (% of normal RBC)	G6PDd not infected RBCs (% of deficient RBC)	G6PDd infected RBCs (% of deficient RBC)	Odds ratio (95% CI), p
Schizont Replicate#1	9,610 (98.0)	192 (2.0)	51,252 (99.6)	219 (0.4)	0.2 (0.18 to 0.26), < 0.001
Schizont Replicate#2	13,821 (97.9)	291 (2.1)	85,019 (99.6)	368 (0.4)	0.2 (0.18 to 0.24), < 0.001
Ring Replicate#1	10,517 (99.1)	97 (0.9)	88,575 (99.7)	223 (0.3)	0.3 (0.21 to 0.35), < 0.001
Ring Replicate#2	8,349 (99.1)	74 (0.9)	90,803 (99.8)	159 (0.2)	0.2 (0.15 to 0.26), < 0.001
Pooled	42,297 (98.5)	654 (1.5)	315,649 (99.7)	969 (0.3)	0.2 (0.18 to 0.22), < 0.001

*CI* confidence interval, *G6PDd* G6PD deficient, *G6PDn* G6PD normal, *RBC* red blood cell

The proportion of G6PD deficient RBCs increased in the second cycle. Since five times more normal RBCs were infected during the first cycle and then subsequently destroyed during schizont rupture, the proportion of normal and deficient cells shifted in favor of deficient cells in vitro. In contrast in in vivo infections, most cells lost from the bloodstream are not parasitized [9–11].

Our flow cytometry-based assay provides a novel approach to quantify the preferential invasion of *P. falciparum* of G6PD normal RBCs compared to deficient cells on a cellular level. The staining agent, DRAQ5, stains malaria parasite DNA irrespective of species, and RBCs are distinguished solely by phenotypic G6PD activity, irrespective of underlying genotype. The presented assay will, therefore, allow quantification of a protective effect for different genetic G6PD variants and against different human pathogenic *Plasmodium* species. How the observed protection from invasion on a cellular level translates to a population-based protective effect of G6PD deficiency against malaria will need to be determined in subsequent field studies.

## Limitations

Our study presents proof-of-concept of a novel approach; however, only includes one heterozygous female and a lab-adapted strain of *P. falciparum.* Whether the developed assay indeed works on different malaria strains and genotypes will have to be confirmed in a broader field trial with blood samples from heterozygous patients with malaria.

## Supplementary Information

**Additional file 1.** Standard operating procedure for G6PD analysis in *Plasmodium*-infected red blood cells.

**Additional file 2.** Flow cytometry data from schizont and ring stages of *P. falciparum* FC27.

**Additional file 3: Figure 1.** Light microscopy images of schizont and ring stages of *Plasmodium falciparum*-infected red blood cells. Legend: A thin blood smear was prepared from *P. falciparum* FC27 culture suspensions supplemented with RBCs from heterozygous female for G6PD gene, and stained with 10% Giemsa. Schizont (A) and Ring developmental stages (B) were observed under 100× oil immersion using an Olympus CX31 light microscope. Scale bar = 10 μm.

**Additional file 4: Figure 2.** Gating strategy of *P. falciparum* FC27 schizont stage for flow cytometry analysis. Legend: RBCs were identified and gated on the forward/side scatter (FS PEAK/SS PEAK) dot plot (panel A). Gating was then applied and vizualised in a side/side scatter (SS INT/SS PEAK) dot plot to select single cells (panel B). The gated single cell population was analysed further in a FL1 histogram to differentiate the G6PD normal and deficient RBC populations (panel C) and FL8/SS PEAK dot plot to differentiate parasitized and non-parasitized RBC populations (panel D). Both gated populations in panel C were visualized in a FL8/SS PEAK dot plot to determine the ratio of infected and non-infected RBCs in the G6PD normal RBCs (panel E) and G6PD deficient RBCs (panel F), respectively, using the gate setting from panel D.

## Data Availability

All relevant data are contained within the manuscript.

## Abbreviations

CI: Confidence interval
CQ: Chloroquine
DNA: Deoxyribonucleic acid
G6PD: Glucose-6-phosphate dehydrogenase
OR: Odds ratio
*P. falciparum*: *Plasmodium falciparum*
PBS: Phosphate-buffered saline
RBC: Red blood cell
WBC: White blood cell
