# Supplementary material for: A fluorometric assay to determine the protective effect of glucose-6-phosphate dehydrogenase (G6PD) against a Plasmodium spp. infection in females heterozygous for the G6PD gene: proof of concept in Plasmodium falciparum
Source: BMC Res Notes. 2022 Feb 22;15:76. doi: 10.1186/s13104-022-05952-1 (PMC8862483; doi:10.1186/s13104-022-05952-1)
Supplement: Supplementary file 1 — Additional file 1. Standard operating procedure for G6PD analysis in Plasmodium-infected red blood cells. [file 13104_2022_5952_MOESM1_ESM.pdf]

Procedure to identify *Plasmodium*-infected deficient and normal red blood cells by flow cytometry

Purpose

To characterise whole blood specimens for intracellular G6PD activity by a flow cytometry-based method as described herein. This method allows observation of mosaic red blood cell populations in specimens from females by looking at the activity of G6PD in individual erythrocytes. This method uses potassium cyanide for the standard colorimetric cyanmethemoglobin analysis.

Scope

This SOP allows observation of mosaic red blood cell populations in specimens from females by looking at the activity of G6PD in individual erythrocytes. This methodology helps establishing a specimen panel for the evaluation of G6PD point-of-care (POC) tests.

Materials required

- Pipettors: p10, p20, p200, p1000, including respective pipette tips
- Centrifuge – It must be placed inside the fume hood
- Ziplock bag and 50mL Gibco bottle (for dry and liquid waste)
- Distilled water
- 50 mL Measuring Cylinder
- 50 mL Gibco bottle and aluminum foil
- 1.7 mL Eppendorf tubes
- DRAQ5™ fluorescent probe, BioLegend®: Cat# 424101, AUSTRALIAN biosearch
- Phosphate Buffered Saline (PBS), Sigma: P4417-100TAB; Dissolve in water
- Sodium Nitrite, Sigma: S2252-500g; Dissolve in water
- Glucose, Aldrich:158968-500g; Dissolve in PBS
- Nile Blue Sulfate, Sigma: N0766-5g; Dissolve in water
- Potassium cyanide, Sigma: 60178-25G.

NOTE: Potassium cyanide is highly poisonous and has to be handled with extreme care in a fume hood only.

- 3% Hydrogen Peroxide, Fluka: 14911-250ml; Dissolve in PBS
- pH 10 buffer solution, Merck: QC1117-250ML
- Hypochlorinate solution (Bleach), Figleaf pool products: UN No. 1791, 125g/L
- Falcon® Round-Bottom Polystyrene Tubes (FACS tube, 5mL), Stemcell: 38007

Definitions

G6PD	Glucose-6-phosphate dehydrogenase is an enzyme in red blood cells that helps the body to metabolize carbohydrates into energy and protects the red blood cells from oxidative stress.
POC	Point-of-care (POC) testing is medical testing that is performed outside of a laboratory setting. POC testing is also known as bedside testing, near-patient testing, remote testing, mobile testing and rapid diagnostics.
SDS	A Safety Data Sheet (SDS), previously called a Material Safety Data Sheet (MSDS), is a document that provides information on the properties of hazardous chemicals and how they affect health and safety in the workplace. An SDS includes information on the identity of the chemical, health and physicochemical hazards, safe handling and storage procedures, emergency procedures, and disposal considerations. The SDS should always be referred to when assessing risks in the workplace.
Usage Register	Spreadsheet used to record the date, quantity, name of person, use and balance of each substance every time it is used. This spreadsheet is kept in the S7 Poison Register.
Register reminder stickers	Bright orange stickers used as a reminder for research staff to complete the usage register each time they use a S7 substance (In this case, Potassium Cyanide).
KCN	KCN is the chemical formula for potassium cyanide. Potassium cyanide is highly toxic and needs to be handled with extreme care in the fume hood only.

Associated Hazards

Biological	Chemicals and / or harmful substances	Environmental	Manual Handling

Procedure – Working with KCN

Setting up the workspace

- Never work alone and/or outside of business hours when using KCN. Using the buddy system, another lab member should always be present and available to assist in the event of a cyanide emergency. Both the user and buddy should have a thorough understanding of safe working guidelines with KCN.
- Always wear double medical grade nitrile gloves and inspect gloves prior to each use. Do not use damaged gloves. Cuffs of the inner gloves need to be tucked under the sleeve of the gown, while outer gloves are worn over the cuff of the gown. Make sure that the cuffs of the gloves go as far up your arms as possible. Use proper glove removal technique (without touching glove's outer surface) to avoid skin contact with KCN.
- Always work with KCN in a fume hood. Turn on the fume hood for work. Check alarms and monitors to indicate proper operation and wipe the benchtop with 70% alcohol. Carry out all work at least 6 inches into the hood from the plane of the sash.
- Clearly label a Ziplock bag and 50 mL Gibco bottle for use as KCN dry and liquid waste container, respectively. KCN waste need to be kept with all KCN vessels in a storage container and locked away.
- Collect key from Laboratory Support Staff to access the S7 storage cabinet. Record the details of use in the usage register. If KCN aliquots or dilutions are to be made, collect the register reminder sticker from Laboratory Support Staff.
- Wrap all KCN vessels with aluminum foil to prevent exposure to light and put a register reminder sticker on.
- After completing work, clean all KCN vessels and pipettes with pH 10 buffer solution, followed by freshly prepared 10% bleach solution. Follow cleaning the pipettes with distilled water and 70% ethanol. Discard the wipes into the dry waste bottle. Set all materials to the side of the hood.
- Wipe the benchtop in the following order with pH 10 buffer solution, freshly prepared 10% bleach, distilled water and 70% ethanol. Discard the wipes and gloves into the dry waste bottle. With a new pair of gloves, place waste bottle/tube, KCN solution and pipettes in a storage container and return it to the S7 storage cabinet. Return the key to Laboratory Support Staff and update details of the KCN usage in the usage register.

Preparing potassium cyanide solution from original sigma vial

- Prepare 38.4 mL of distilled water in a measuring cylinder as solvent for KCN solution.
- Set up the work area as described in the section "Setting up the workspace".
- Prepare 10 M KCN solution by first adding 2 mL of distilled water to the potassium cyanide sigma vial and mix well by pipetting up and down. Transfer the liquid substance to a clearly labelled 50 mL Gibco bottle.
- Repeat the above step.
- Add the remaining distilled water to the Gibco bottle and mix well by swirling the tube until the substance is thoroughly dissolved.
- Clean up the area.

Procedure – G6PD flow cytometry assay

- Prepare staining solution by adding 83.75 µL of PBS and 6.25 µL of DRAQ5™ fluorescent probe at working concentration (100 mM) in an Eppendorf tube.
- Add 10 µL of 50% haematocrit RBC/iRBC suspension to each tube and incubate at room temperature for 20 minutes.
- Wash samples two times in 100 µL PBS with centrifugation at 3,000 rpm for 1 minute. After each centrifugation step, discard supernatant into an appropriate waste container.
- Resuspend the samples in 100 µL PBS.
- Add 100 µL of 0.125 M sodium nitrite and incubate at room temperature for 20 minutes.
- Wash samples three times in 100 µL PBS with centrifugation at 3,000 rpm for 1 minute. After each centrifugation step, discard supernatant into an appropriate waste container.
- Resuspend the samples in 100 µL PBS.
- Add 18 µL of 0.28 M glucose and 6 µL of 0.01% Nile blue sulfate to each tube.
- Incubate samples at 37°C for 90 minutes in an aerobic environment, with the lids open.
- Place incubated samples and all required materials in the fume hood.
- Working at least 6 inches into the hood from the plane of the sash, prepare 125 µL of 0.4 M KCN solution by adding 5 µL 10M KCN solution (from Section above) and 120 µL distilled water in a clearly labelled 1.7 mL Eppendorf tube.

NOTE: Potassium Cyanide is highly poisonous and must be handled with extreme care in a fume hood only.

- Add 2.5 µL 0.4 M potassium cyanide to each sample and incubate at room temperature for 5 minutes.
- Add 5 µL of each sample to 100 µL 0.3% hydrogen peroxide. Discard the remaining sample into liquid waste container and the Eppendorf tube into dry waste container.
- Close the lids tightly and agitate vigorously by hand.
- Wash samples two times in 500 µL PBS with centrifugation at 3,000 rpm for 3 minutes. After each centrifugation step, discard supernatant into the liquid waste container.
- Resuspend cells in 500 µL PBS in FACS tubes for analysis on Gallios flow cytometer (Beckman Coulter, Brea, CA, USA) using FL1 (533 ± 30 nm bandpass filter) and FL8 (775 long pass filter).
