# Supplementary material for: A fluorometric assay to determine the protective effect of glucose-6-phosphate dehydrogenase (G6PD) against a Plasmodium spp. infection in females heterozygous for the G6PD gene: proof of concept in Plasmodium falciparum
Source: BMC Res Notes. 2022 Feb 22;15:76. doi: 10.1186/s13104-022-05952-1 (PMC8862483; doi:10.1186/s13104-022-05952-1)
Supplement: Supplementary file 2 — Additional file 2. Flow cytometry data from schizont and ring stages of P. falciparum FC27. [file 13104_2022_5952_MOESM2_ESM.pdf]

201006 Neat E.Rings FC27 156
G6PD FACS - Imported

[Ungated] FS PEAK / SS PEAK

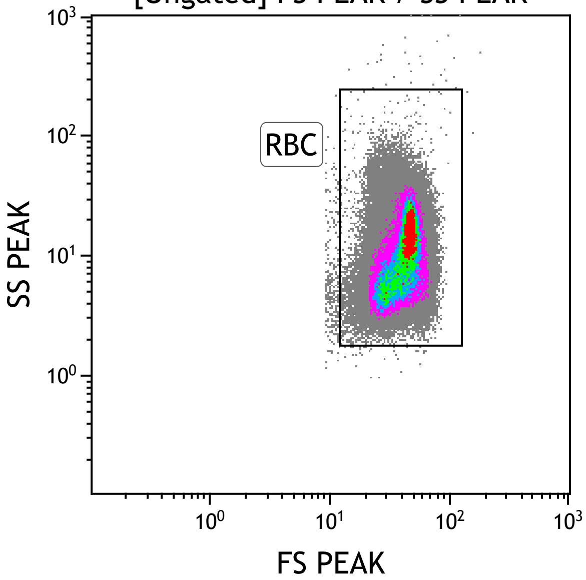

Gate	Number	%Total	%Gated
All	100,000	100.000	100.000
RBC	99,624	99.624	99.624

[RBC] SS INT / SS PEAK

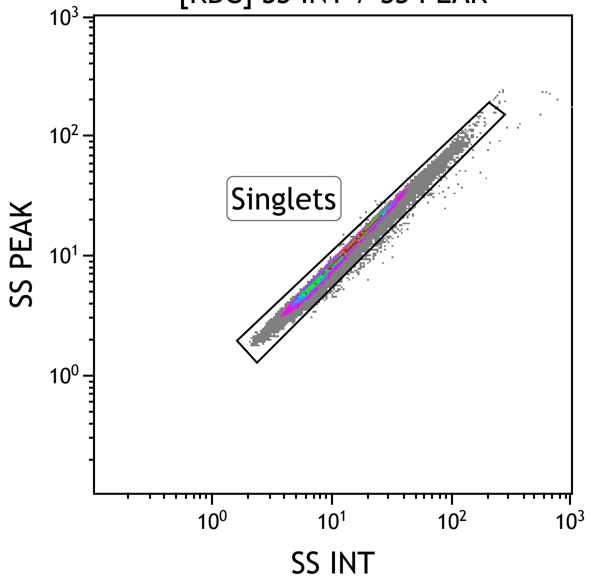

Gate	Number	%Total	%Gated
All	99,624	99.624	100.000
Singlets	99,412	99.412	99.787

[Singlets] FL1 INT

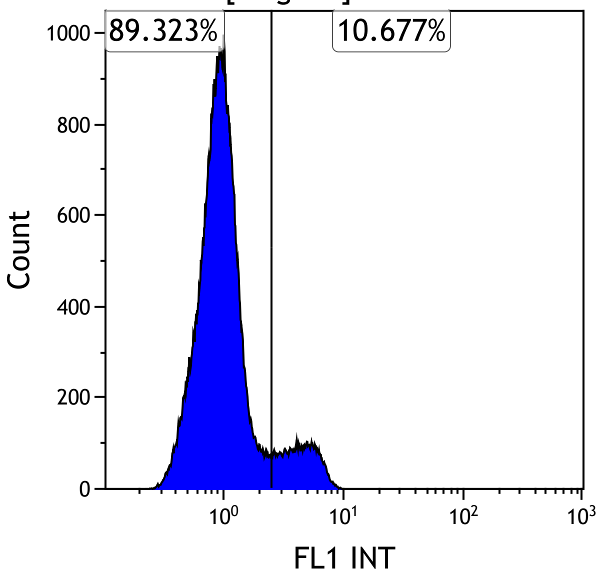

Gate	Number	%Gated
All	99,412	100.000
G6PD-	88,798	89.323
G6PD+	10,614	10.677

[Singlets] FL8 INT / SS PEAK

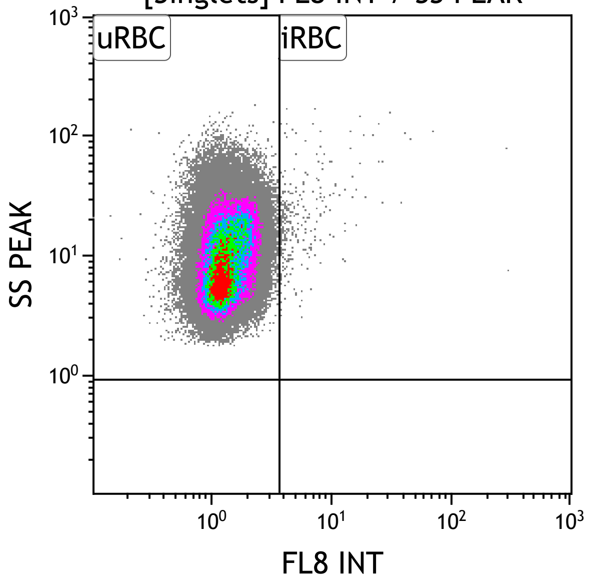

Gate	Number	%Gated
All	99,412	100.000
iRBC	320	0.322
uRBC	99,092	99.678

201006 Neat E.Rings FC27 156
G6PD FACS - Imported

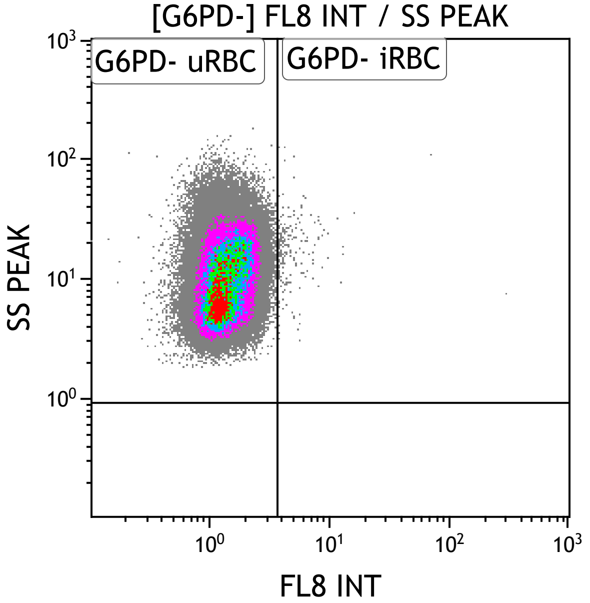

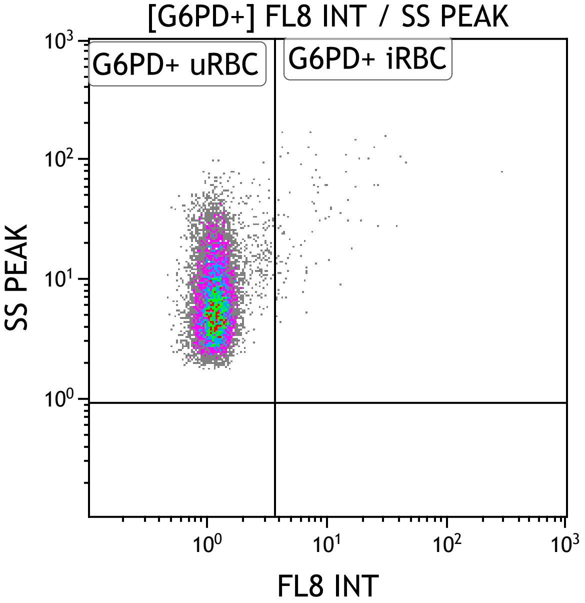

Gate	Number	%Gated
All	88,798	100.000
G6PD- iRBC	223	0.251
G6PD- uRBC	88,575	99.749

Gate	Number	%Gated
All	10,614	100.000
G6PD+ iRBC	97	0.914
G6PD+ uRBC	10,517	99.086

201006 Neat E.Rings FC27 167

G6PD FACS - Imported

[Ungated] FS PEAK / SS PEAK

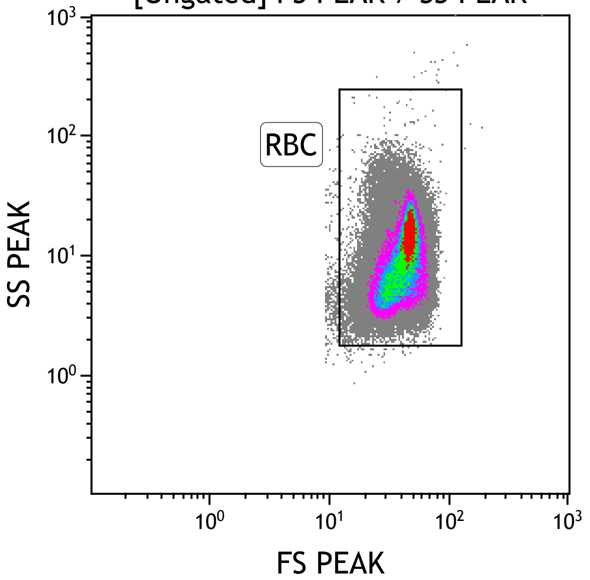

Gate	Number	%Total	%Gated
All	100,000	100.000	100.000
RBC	99,639	99.639	99.639

[RBC] SS INT / SS PEAK

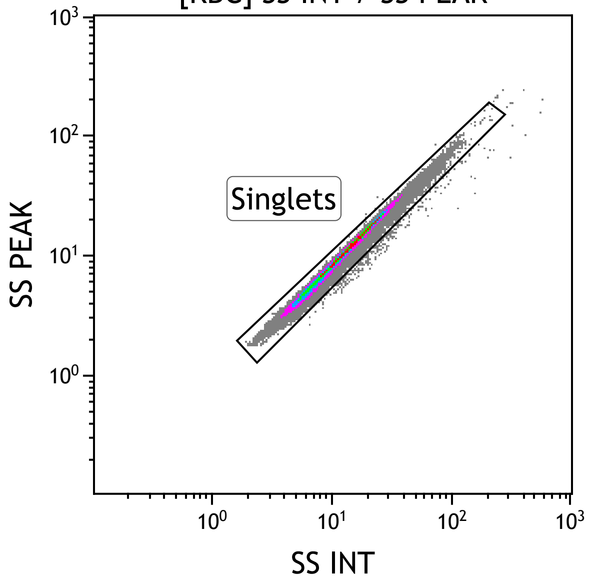

Gate	Number	%Total	%Gated
All	99,639	99.639	100.000
Singlets	99,385	99.385	99.745

[Singlets] FL1 INT

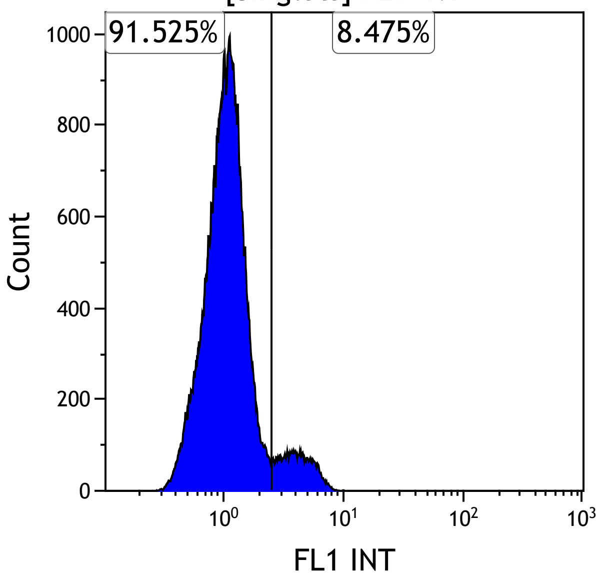

Gate	Number	%Gated
All	99,385	100.000
G6PD-	90,962	91.525
G6PD+	8,423	8.475

[Singlets] FL8 INT / SS PEAK

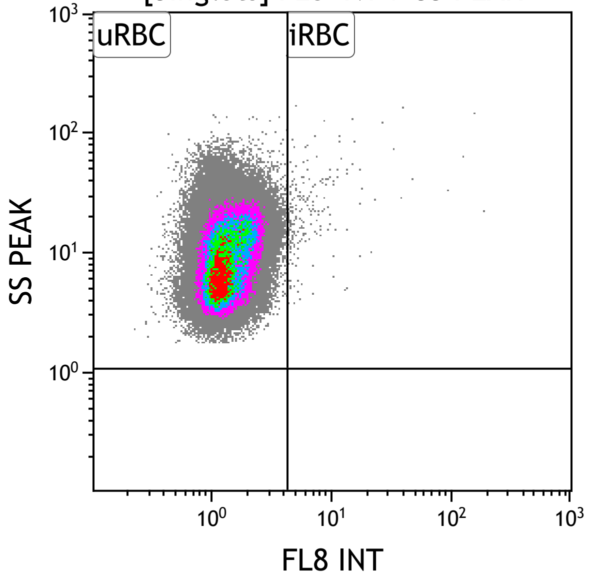

Gate	Number	%Gated
All	99,385	100.000
iRBC	232	0.233
uRBC	99,153	99.767

201006 Neat E.Rings FC27 167
G6PD FACS - Imported

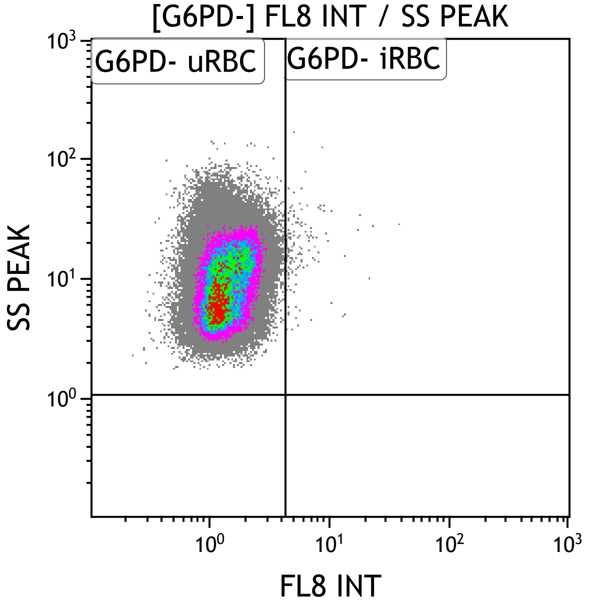

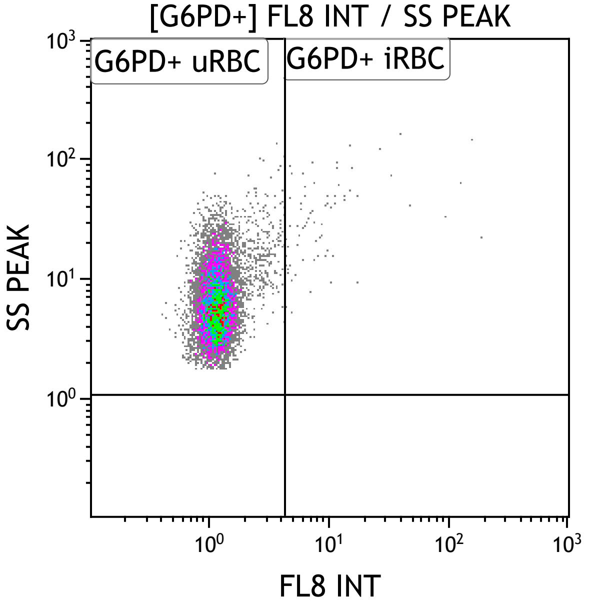

Gate	Number	%Gated
All	90,962	100.000
G6PD- iRBC	159	0.175
G6PD- uRBC	90,803	99.825

Gate	Number	%Gated
All	8,423	100.000
G6PD+ iRBC	74	0.879
G6PD+ uRBC	8,349	99.121

201006 Neat Schiz FC27 159
G6PD FACS - Imported

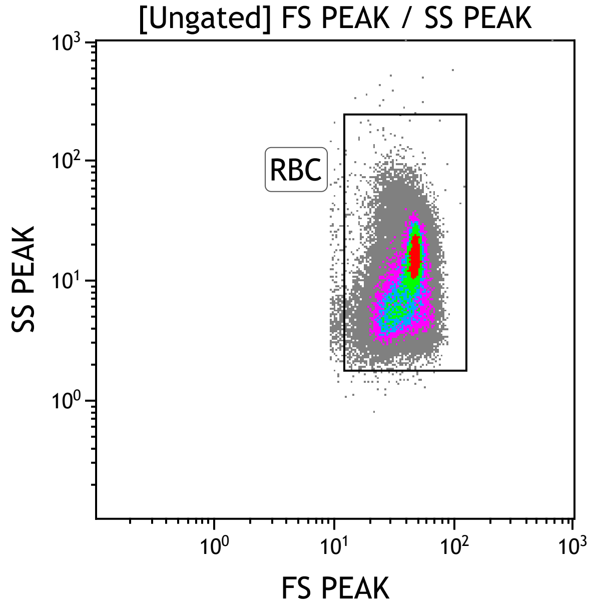

Gate	Number	%Total	%Gated
All	61,774	100.000	100.000
RBC	61,370	99.346	99.346

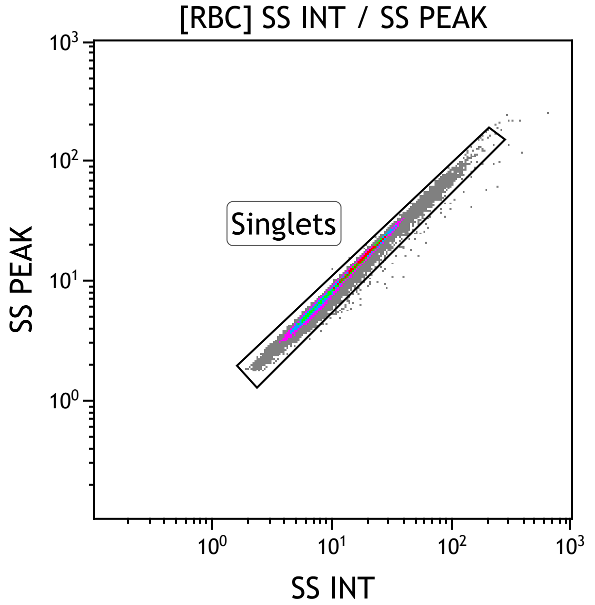

Gate	Number	%Total	%Gated
All	61,370	99.346	100.000
Singlets	61,273	99.189	99.842

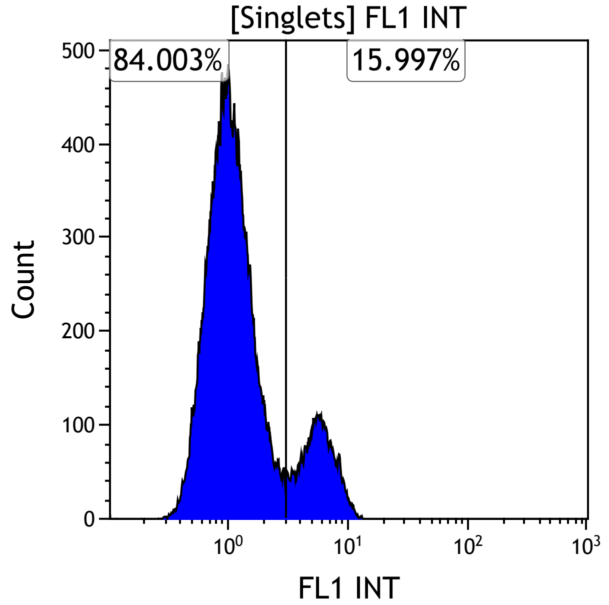

Gate	Number	%Gated
All	61,273	100.000
G6PD-	51,471	84.003
G6PD+	9,802	15.997

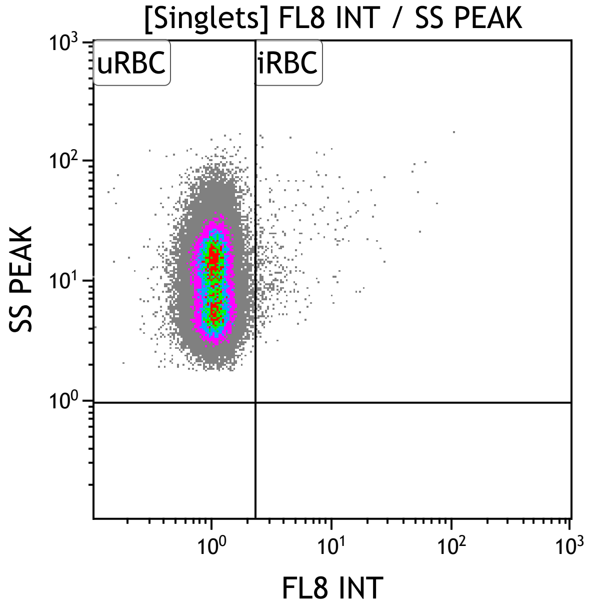

Gate	Number	%Gated
All	61,273	100.000
iRBC	411	0.671
uRBC	60,862	99.329

201006 Neat Schiz FC27 159
G6PD FACS - Imported

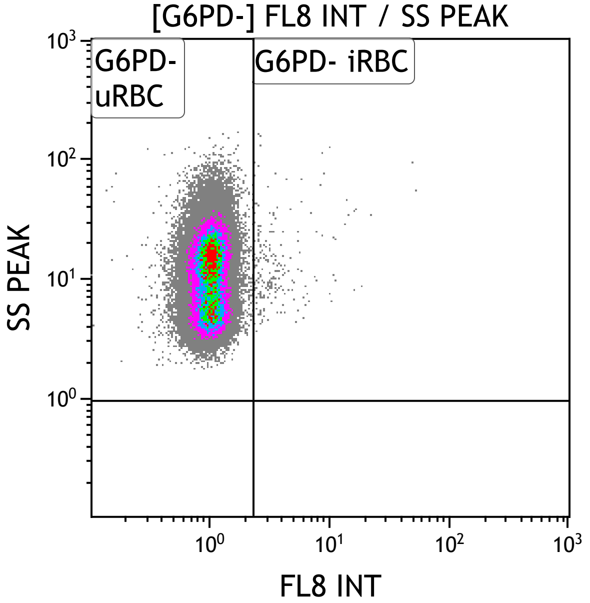

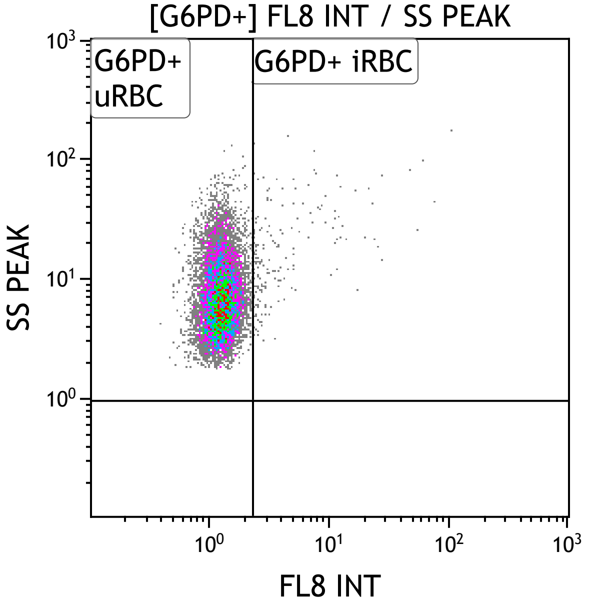

Gate	Number	%Gated
All	51,471	100.000
G6PD- iRBC	219	0.425
G6PD- uRBC	51,252	99.575

Gate	Number	%Gated
All	9,802	100.000
G6PD+ iRBC	192	1.959
G6PD+ uRBC	9,610	98.041

201006 FC27 Schizonts Neat 152
G6PD FACS - Imported

[Ungated] FS PEAK / SS PEAK

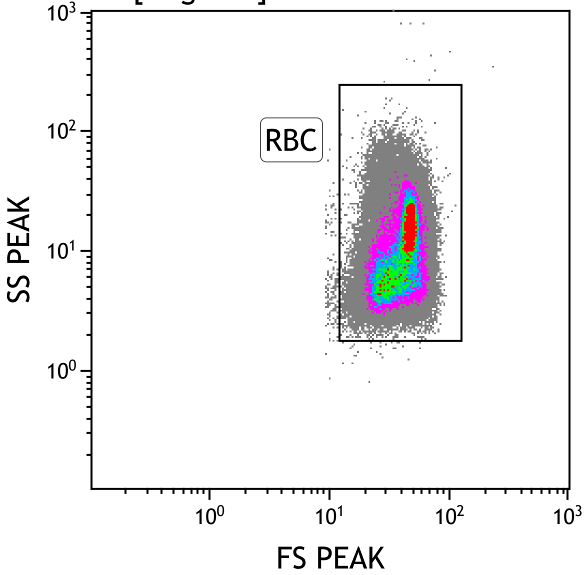

Gate	Number	%Total	%Gated
All	100,000	100.000	100.000
RBC	99,772	99.772	99.772

[RBC] SS INT / SS PEAK

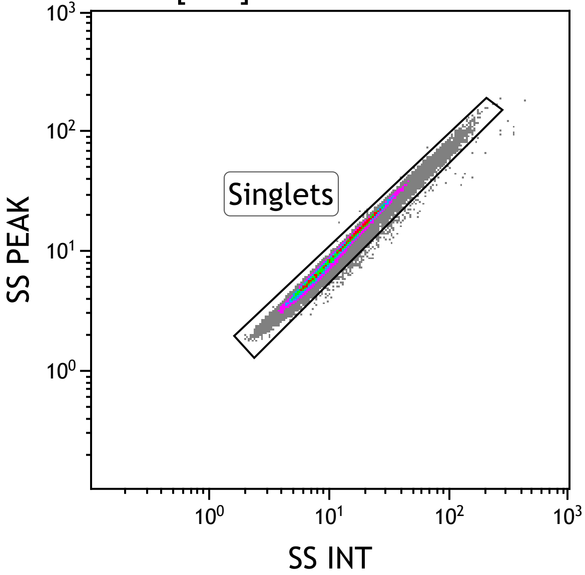

Gate	Number	%Total	%Gated
All	99,772	99.772	100.000
Singlets	99,499	99.499	99.726

[Singlets] FL1 INT

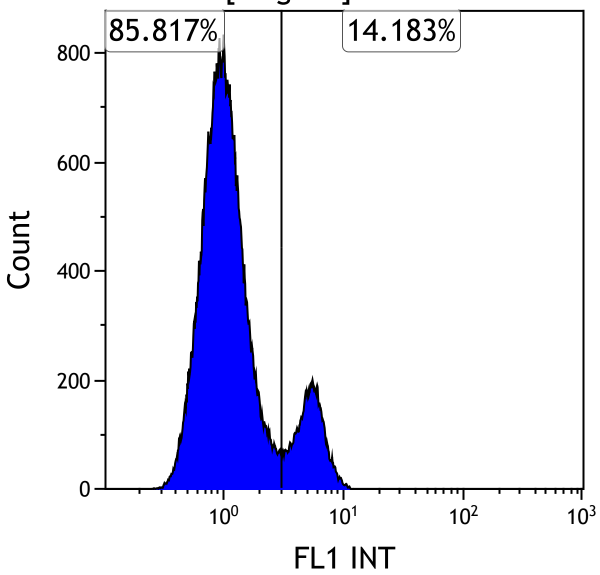

Gate	Number	%Gated
All	99,499	100.000
G6PD-	85,387	85.817
G6PD+	14,112	14.183

[Singlets] FL8 INT / SS PEAK

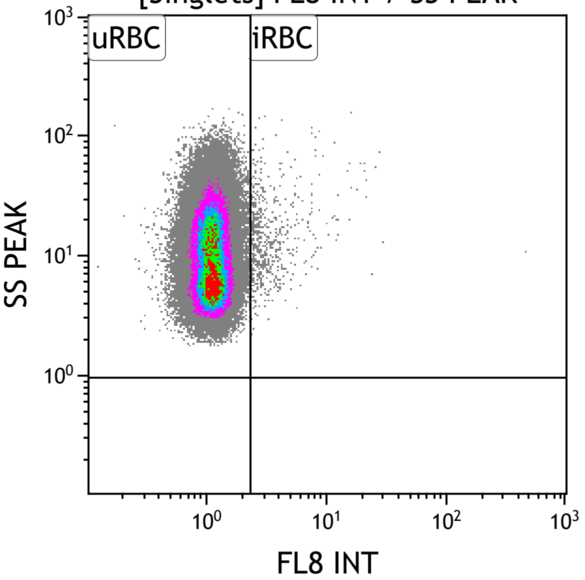

Gate	Number	%Gated
All	99,499	100.000
iRBC	659	0.662
uRBC	98,840	99.338

201006 FC27 Schizonts Neat 152
G6PD FACS - Imported

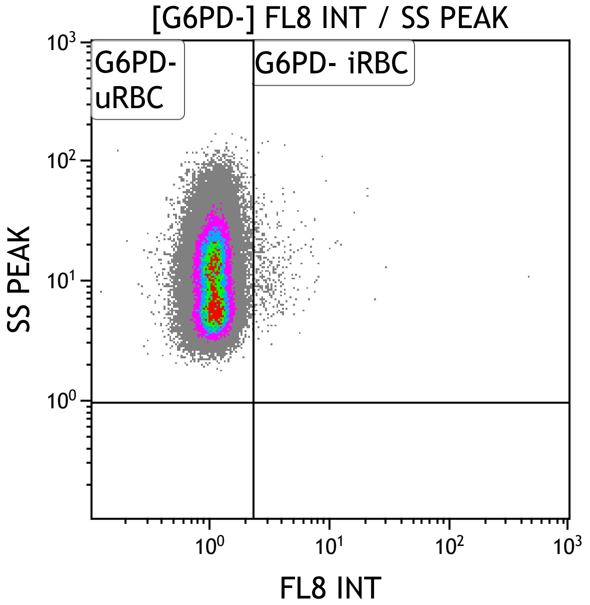

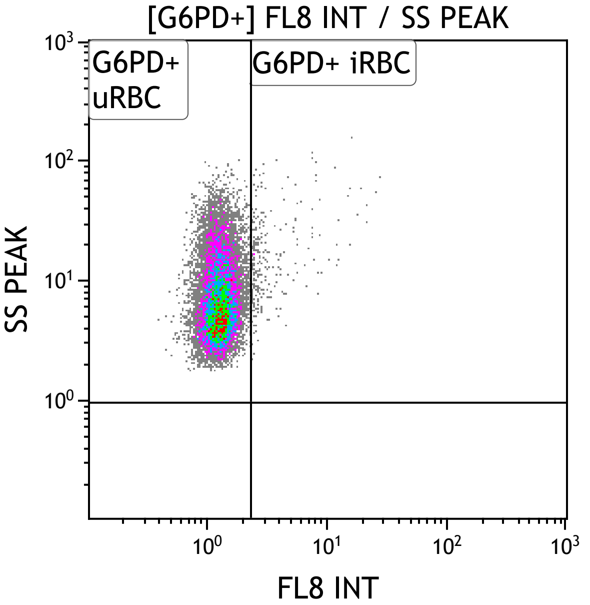

Gate	Number	%Gated
All	85,387	100.000
G6PD- iRBC	368	0.431
G6PD- uRBC	85,019	99.569

Gate	Number	%Gated
All	14,112	100.000
G6PD+ iRBC	291	2.062
G6PD+ uRBC	13,821	97.938
