# Supplementary figures and images for: A fluorometric assay to determine the protective effect of glucose-6-phosphate dehydrogenase (G6PD) against a Plasmodium spp. infection in females heterozygous for the G6PD gene: proof of concept in Plasmodium falciparum
Source: BMC Res Notes. 2022 Feb 22;15:76. doi: 10.1186/s13104-022-05952-1 (PMC8862483; doi:10.1186/s13104-022-05952-1)

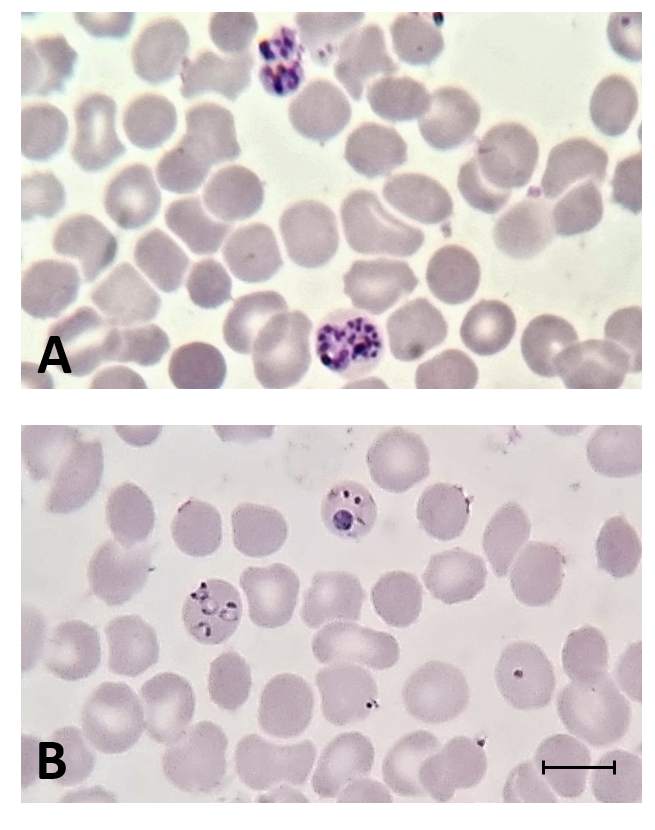

Supplement: Supplementary file 3 — Additional file 3: Figure 1. Light microscopy images of schizont and ring stages of Plasmodium falciparum-infected red blood cells. Legend: A thin blood smear was prepared from P. falciparum FC27 culture suspensions supplemented with RBCs from heterozygous female for G6PD gene, and stained with 10% Giemsa. Schizont (A) and Ring developmental stages (B) were observed under 100× oil immersion using an Olympus CX31 light microscope. Scale bar = 10 μm. [file 13104_2022_5952_MOESM3_ESM.png]

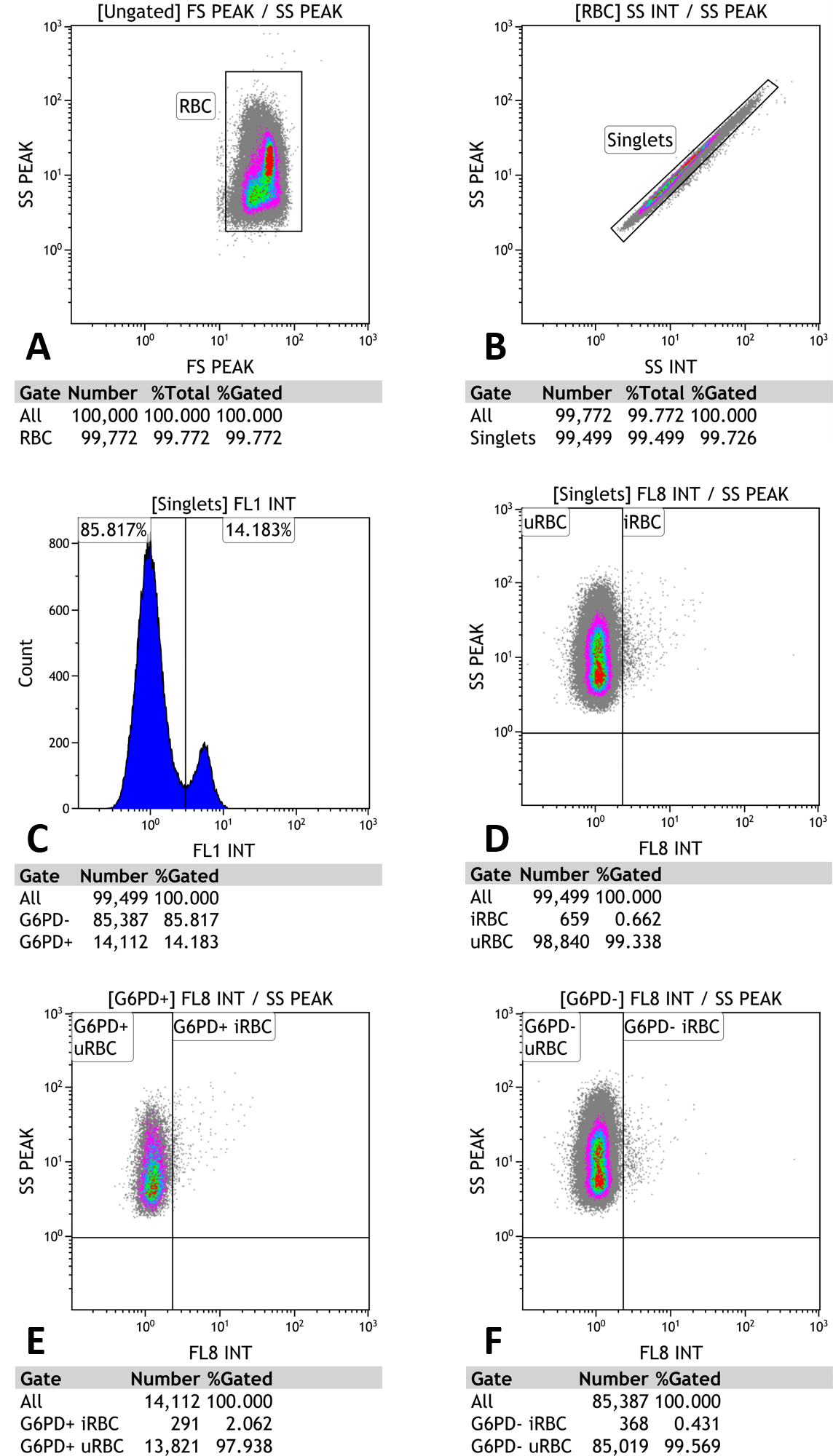

Supplement: Supplementary file 4 — Additional file 4: Figure 2. Gating strategy of P. falciparum FC27 schizont stage for flow cytometry analysis. Legend: RBCs were identified and gated on the forward/side scatter (FS PEAK/SS PEAK) dot plot (panel A). Gating was then applied and vizualised in a side/side scatter (SS INT/SS PEAK) dot plot to select single cells (panel B). The gated single cell population was analysed further in a FL1 histogram to differentiate the G6PD normal and deficient RBC populations (panel C) and FL8/SS PEAK dot plot to differentiate parasitized and non-parasitized RBC populations (panel D). Both gated populations in panel C were visualized in a FL8/SS PEAK dot plot to determine the ratio of infected and non-infected RBCs in the G6PD normal RBCs (panel E) and G6PD deficient RBCs (panel F), respectively, using the gate setting from panel D. [file 13104_2022_5952_MOESM4_ESM.png]
